# Commentary: Synergy Between Beta-Lactams and Lipo-, Glyco-, and Lipoglycopeptides is Independent of the Seesaw Effect in Methicillin-Resistant *Staphylococcus aureus*

**DOI:** 10.3389/fmolb.2021.774021

**Published:** 2021-10-07

**Authors:** Emily M. Meredith, Andrew D. Berti

**Affiliations:** ^1^ Department of Pharmacy Practice, Eugene Applebaum College of Pharmacy and Health Sciences, Wayne State University, Detroit, MI, United States; ^2^ Department of Biochemistry, Microbiology and Immunology, College of Medicine, Wayne State University, Detroit, MI, United States

**Keywords:** seesaw, synergy, combination thera py, MRSA, methicillin-resistant staphylococcus aureus, lipidomics

## Commentary Text

Appropriate use of antimicrobials is an issue of much debate in contemporary clinical practice (Rashidzada et al., 2021). Treatment of some pathogens such as tuberculosis or human immunodeficiency virus often requires combinations of antimicrobials whereas antimicrobial stewardship encourages a reduction in unnecessary antimicrobial exposure to prevent the emergence of antimicrobial resistance. Treatment of complicated methicillin-resistant *staphylococcus aureus* (MRSA) infection sits at the interface of these two imperatives. Combination treatment pairing a beta lactam with a glycopeptide (*e.g.*, vancomycin [VAN]), a lipopeptide (*e.g.*, daptomycin [DAP]) or a lipoglycopeptide (*e.g.*, telavancin, dalbavancin [DAL] or oritavancin) has gained support in the treatment of MRSA due to synergistic *in vitro* and clinical evidence (Alosaimy et al., 2021). However, the mechanism for this therapeutic enhancement is poorly understood as beta lactams other than ceftaroline have minimal direct activity against MRSA. Hypotheses have been proposed based on several distinct observations. As MRSA develops resistance to the non-beta lactam antibiotic (*i.e.*, the MIC increases), the MIC to beta lactams frequently decreases in a phenomenon known as the “seesaw effect” (Ortwine et al., 2013). In the absence of resistance development, the presence of subinhibitory non-beta lactam antibiotic can lower the MIC of beta lactams or enhance the extent or rate of bacterial killing (“synergy”) (Tran and Rybak, 2018). Finally, the presence of beta lactams can delay or prevent the emergence of resistance to the non-beta lactam antibiotic (Berti et al., 2012). The physiological mechanisms, the inter-relatedness and the relative contributions of these phenomena to clinical success are unclear.

The article by Zhang et al. explores the interdependency of these phenomena. The authors derived strains with elevated MICs to VAN, DAP and DAL via subinhibitory serial passage. Derived strains passaged in VAN or DAP exhibited the seesaw effect whereas the strain passaged in DAL did not. This was particularly evident with nafcillin (NAF) with a 32-fold reduction in NAF MIC for strains that had been passaged in VAN or DAP but no change in NAF MIC for strains that had been passaged in DAL. Similar results were observed with cefalexin (LEX) but not with other beta lactams tested indicating distinctions in the seesaw effect depending on the beta lactam selected. In contrast to the MIC results, enhanced killing was observed in all derived strains including those passaged in DAL. Notably, beta lactams such as ceftriaxone and cefoxitin with minimum change to the MIC in derived strains were particularly effective at increasing the killing activity in combination. This clearly uncouples the observations of seesaw and synergy such that the presence of one does not necessarily imply the presence of the other.

The authors then explore potential physiological changes associated with therapeutic enhancement. Analysis of the membrane phospholipids in general demonstrated an increase in free fatty acid and cardiolipin content and a decrease in lysylphosphatidylglycerol content when exposed to a “synergistic” beta lactam. Of note, none of the derived strains contain mutations in *mprF* known to modulate membrane lysylphosphatidylglycerol content (Ernst and Peschel, 2019). Therefore, the benefits of beta lactam exposure do not require a baseline enrichment in lysylphosphatidylglycerol due to *mprF* gain-in-function. However, strains that exhibited the most pronounced decreases in lysylphosphatidylglycerol content upon beta lactam exposure had mutations in the *walKR* and *vraSRT* systems that regulate cell envelope homeostasis and indirectly modulate lysylphosphatidylglycerol content (Pietiäinen et al., 2009; Kuroda et al., 2019). Together, these findings support links between beta lactam exposure and modulation of membrane lipid physiology.

Limitations from this study include the use of a single strain; while N315 does demonstrate the distinctions between the phenomena, it would be strengthened if more relevant stains were used. Another limitation includes the use of inconsistent concentrations of beta lactam antibiotics. Although necessary to maintain subinhibitory concentrations, the different relative amounts of these beta lactams challenge the interpretation of the data collected. We do note, however, that each of the concentrations used was physiologically-relevant and would be consistent with the expected serum concentrations at some point during a standard dosing interval. Although this *in vitro* study alone will not change current practice, it elucidates how combination therapy may be beneficial and reinforces that drug MICs alone not always the best predictor of antimicrobial efficacy.
